# Cerebrovascular Accident Manifesting as Altered Mental Status During a Telehealth Visit: Preoperative Evaluation in the COVID-19 Era

**DOI:** 10.7759/cureus.13812

**Published:** 2021-03-10

**Authors:** Cecilia Canales, Lydiesther Martinez, Nirav Kamdar

**Affiliations:** 1 Anesthesiology & Perioperative Medicine, University of California, Los Angeles (UCLA) Health, Los Angeles, USA

**Keywords:** tele-stroke, preoperative, preoperative screening

## Abstract

We present the case of a 91-year-old patient scheduled for a preoperative telehealth evaluation who was found to have altered mental status from an acute stroke. Her care, if delayed, could have caused permanent morbidity during the coronavirus disease 2019 (COVID-19) pandemic. This case highlights the digital leap the pandemic spurred: 1. telehealth in the elderly, 2. meaningful history and physical during telehealth visit, 3. family engagement and education, and 4. meaningful impact on patient outcomes.

## Introduction

Preoperative evaluations are of the utmost importance to safe and effective intra- and post-operative management. Patients with complex diseases and who are at risk for precarious outcomes often benefit from a preoperative evaluation from a preoperative specialist [1]. With increasing frequency, medical centers and anesthesiology departments are implementing preoperative evaluation clinics to improve quality of care, reduce costs, reduce same-day surgical cancellations, and optimize patients for surgery [1-4].

The adoption of telehealth for preoperative evaluation clinics has gained some traction to reach patients who may not otherwise present to a clinic [5,6]. Patients who require multiple short visits for preoperative optimization, such as those enrolled in smoking cessation [7] or weight loss [8] programs have shown benefit from telehealth visits. Telehealth also plays a critical role in the preoperative clinic for those who require titration of medications and who routinely update health information into mobile applications tied to their electronic medical record, such as patients with pheochromocytomas [9] or patients with uncontrolled diabetes [10] prior to surgery [6].

When the severe acute respiratory syndrome-coronavirus 2 (SARS-CoV-2) infection and risks of the coronavirus disease 2019 (COVID-19) pandemic became apparent, it forced healthcare providers and institutions to adopt telehealth initiatives and gave new impetus for its growth and applications [11-13]. Telehealth allowed for the ability of continuum of care, providing a needed resource to our patients while reducing patient and healthcare provider exposure. While many elective non-emergent cases were postponed or cancelled worldwide, surgeries that were scheduled were often for patients requiring the greatest optimization prior to surgery [14]. Although surgical volumes were low during the time of COVID-19, perioperative telehealth became the portal to maintain the health of our most vulnerable patients. We present a case report of a preoperative telehealth evaluation of a patient during the COVID-19 pandemic with altered mental status, and how the preoperative telehealth visit changed patient care to prevent catastrophic neurologic injury. Written authorization was obtained by the patient’s durable power of attorney and exemption was granted from the local institutional review board. 

## Case presentation

Several weeks into the COVID-19 pandemic, we encountered a 91-year-old woman during a telehealth preoperative evaluation for total abdominal hysterectomy for management of endometrial adenocarcinoma. She was evaluated by her primary care physician two days prior to the telehealth visit and reported to be neurocognitively intact. Except for incontinence, her past medical history was unremarkable, capable of all activities of daily living. The patient lived in an independent living facility with meal service. She administered her own medications and, prior to COVID-19 isolation requirements, regularly exercised, shopped, and socialized with residents at the facility. A recent electrocardiogram showed sinus bradycardia 59 beats per minute with normal axis, incomplete right bundle branch block, absent pathologic Q waves or ST segment changes. Laboratory tests on the day of her primary care visit revealed mildly elevated potassium (5.5 mmol/L) [normal range 3.6-5.2 mmol/L] and calcium (11.1 mg/dL) [normal range 8.5-10.2 mg/dL] though repeat labs were within normal range. 

On initiation of the telehealth visit, the patient’s daughter answered and noted she arrived early to assist her mother with the technology for the telehealth visit. The daughter, who visits often, expressed concern because her mother appeared altered, babbling, and pointing incoherently, which was different from her baseline. The anesthesiology resident attempted to confirm the patient identity, but she was unable to provide name or birthdate, though her daughter confirmed both identifiers. 

Although the daughter mentioned unusual behavior to the staff of the long-term care facility, the last known normal baseline could not be ascertained. Further history revealed the patient currently did not have any vaginal bleeding from endometrial cancer, had no recent trauma, had no constitutional symptoms, no fever, chills, shortness of breath, chest pain, or weakness. The patient was in no acute distress, although confused, but was able to answer questions and follow simple commands. The daughter contemplated transporting her mother to the emergency room, but ultimately decided against it given her fear of her mother being at increased risk in a hospital due to her elevated age and the COVID-19 pandemic. 

A cursory neurologic and stroke assessment was performed, specifically assessing cranial nerve VII (smile, raise eyebrows, frown, puff out their cheeks while looking for asymmetry), asking the patient to repeat a simple sentence (the sky is blue), asking the patient to extend arms at 90 degrees and assessing for drift, and asking whether she recognized the person standing next to her (her daughter). A mini-cognition questionnaire was attempted but the patient could not cooperate with three-word recall. The daughter provided her mother with a pen and paper, and the patient was asked to draw a clock with hands indicating the time “10 past 11.” The patient spatially drew a circular clock, but ultimately drew a highly inaccurate image. Given the concerning history and physical the resident paged the attending who joined the telemedicine video for further assistance.

The daughter was advised to call 911 or take her mother to the nearest emergency room given presenting symptoms of altered mental status and its wide differential including urinary tract infection given her known incontinence, metabolic derangement given recently elevated calcium and potassium, infection including meningitis or encephalitis, cerebral vascular accident or transient ischemic attack, unwitnessed fall, or polypharmacy. The daughter hesitated, citing the COVID-19 pandemic, but was further counseled given the patient was exhibiting symptoms far from baseline, which warranted further workup. The following day the resident followed up and found the patient was hospitalized after workup was consistent with acute cortical and subcortical infarct involving the posterior right temporal lobe. The patient’s mental status continued to wax and wane and she exhibited expressive aphasia. While hospitalized, the patient underwent stroke workup including imaging, echocardiogram, and carotid ultrasound. After five days the patient was discharged to a physical therapy facility and started on anticoagulation for newly diagnosed atrial fibrillation. The patient’s surgery was postponed given significant recovery, and preoperative evaluation was rescheduled.

Her surgery was performed approximately seven weeks after initial consultation. On the day of surgery, her cranial nerve exam was unremarkable, without residual upper or lower motor or sensory deficit. The patient was oriented to person, place, and date, but when the mini-cognition questionnaire was re-administered, she scored 0/5 without much improvement in her clock drawing. 

## Discussion

Our case of a 91-year-old patient who presented for a preoperative telehealth evaluation with altered mental status, which proved to be a large watershed stroke, highlights the importance of telehealth assessments to achieve meaningful and high-quality patient care. At the core of every anesthesiologist is a physician who straddles the line between a surgical and medical practice. During the preoperative evaluation we must be able to anticipate surgical technique, while evaluating patient-specific co-morbidities and advancement of disease. Beyond an airway assessment, preoperative evaluations may be the first time a new abnormality is appreciated, such as a cardiac murmur. In our case, the fundamentals of a neurological exam and routine administration of neurocognitive testing in our elderly patients significantly impacted patient care. This case highlights how the fundamental medical training anesthesiologists received in internal medicine and critical care are essential throughout the perioperative physician’s career.

Although reports show the value added of a preoperative clinic in improving healthcare delivery, reducing surgical cancellation, and patient satisfaction [1-4], healthcare institutions and even departments of anesthesiology have been slow to invest the time, space, and financial resources to run a preoperative evaluation center. Even fewer still have invested in the digital infrastructure for telehealth preoperative evaluations. The investment in preoperative evaluation centers and telehealth, in general, have proven to be essential for patient care as the SARS-CoV-2 infection spread across the world, forcing isolation precautions and a reduction in in-person clinic visits. 

COVID-19 has become a catalyst to transform digital health into a 21st-century resource. For years, healthcare systems and providers have been moving the technology forward incrementally but faced barriers including billing, privacy, funding for infrastructure and support, and patient reluctance to adopt the technology. Within a few weeks of the pandemic, attitudes and policy for telehealth shifted and thus allowed patients to stay home while receiving care and health education. These home visits allowed patients to communicate with their providers virtually, helping to decrease the risk of SARS-CoV-2 infection spread across our vulnerable patient populations, and reduce risk of transmission to our healthcare providers and support staff. Although the COVID-19 pandemic was an impetus for widespread telehealth implementation, post-COVID-19 telehealth is likely to remain. Beyond COVID-19, telehealth gives additional opportunities to provide meaningful patient care. In fact, telehealth can expand services, leading to more personalized patient care where the provider has purview to a patient’s living, sleeping, and social conditions, which all inform patient care. Telehealth clearly has the capacity to play an integral role in healthcare delivery, though there are many instances where in-person health services are more appropriate, such as features of a physical exam that technology has not yet allowed a replacement. For instance, in our neurologic exam we were unable to measure symmetry in handgrip or evaluate pupil reaction. 

An unintended consequence of the COVID-19 pandemic was a decrease in emergency room visits and admissions for common medical urgencies including myocardial infarctions and strokes [15]. Although the incidence of these cases is unlikely to have diminished during the COVID-19 era, our case underscores that patients and families are hesitant to seek medical treatment due to the fear of infection exposure. Once again, the value of the perioperative physician in health counseling and education, building rapport and trust with the family and their loved ones, is indispensable. Further, our follow up ensuring the patient was safe and evaluated at a hospital, and communication with the referring surgeon to keep them apprised of the stroke and recovery, all ensure high-quality, safe, care.

## Conclusions

Telehealth has the ability to meaningfully impact perioperative patient care by providing quality, cost-effective healthcare services especially to vulnerable patients including aging populations. Telehealth cannot take the place of all visits, just as in this case, our patient required in-person emergent care. However, if it were not for the telehealth pre-operative visit, this patient may have delayed care with catastrophic results. In the wake of COVID-19, there is increased awareness of telehealth services, and perioperative physicians should capitalize on this opportunity to provide patient-centered quality care.
